# Fear conditioning depends on the nature of the unconditional stimulus and may be related to hair levels of endocannabinoids

**DOI:** 10.1111/psyp.14297

**Published:** 2023-03-23

**Authors:** Luke J. Ney, David S. Nichols, Ottmar V. Lipp

**Affiliations:** ^1^ School of Psychology and Counselling Queensland University of Technology Brisbane Queensland Australia; ^2^ Central Science Laboratory University of Tasmania Hobart Tasmania Australia

**Keywords:** electrodermal responding, endocannabinoids, fear conditioning, replicability

## Abstract

The replicability of fear conditioning research has come under recent scrutiny, with increasing acknowledgment that the use of differing materials and methods may lead to incongruent results. Direct comparisons between the main two unconditional stimuli used in fear conditioning – an electric shock or a loud scream—are scarce, and yet these stimuli are usually used interchangeably. In the present study, we tested whether a scream, a shock, or an unpredictable combination of the two affected fear acquisition, extinction, and return of fear amongst healthy participants (*N* = 109, 81 female). We also collected hair samples and tested the relationship between fear conditioning and hair endocannabinoid levels. Our findings suggest that, although subjective ratings of pleasantness, arousal, and anxiety were similar regardless of the unconditional stimuli used, skin conductance responses were significantly lower for stimuli paired with the scream compared to a shock alone. Further, reducing the predictability of the unconditional stimulus reduced habituation of skin conductance responses during acquisition and reacquisition, but did not produce stronger conditioning compared to shock alone. Exploratory analyses suggested that hair endocannabinoids were associated with overall physiological arousal during fear conditioning, as well as higher return of fear to the threat cue, but not to the safety cue. These findings have multiple implications for the design and replicability of fear conditioning research and provide the first evidence for an association between hair levels of endocannabinoids and human fear conditioning.

## Abstract 2

This study shows for the first time that reducing the predictability of the nature of the unconditional stimuli does not increase human fear conditioning. Hair endocannabinoids were also measured in conjunction with conditioning, and preliminary results suggest that 2‐AG may be associated with fear renewal.

## INTRODUCTION

Fear conditioning is an important experimental paradigm that seeks to elicit the learning and memory processes underlying anxiety and trauma‐related disorders (Bouton et al., 2020; Craske et al., 2014; Vervliet et al., 2013; Zuj et al., 2016). Fear conditioning involves experimentally modeling fear learning during an ‘acquisition’ phase, where a neutral stimulus (e.g., a picture of an animal) is repeatedly paired with an unconditional stimulus (e.g., an electric shock, US). After being paired with the US, the neutral stimulus is now a conditional stimulus (CS+) as it has been conditioned to elicit a physiological response, even in absence of the US. A second neutral stimulus (the CS−) will often serve as a control and a safety signal during a fear conditioning experiment, and it is not followed by the US at any stage. Responding is usually measured using skin conductance responses (SCRs), though other outcome measures such as subjective ratings of CS pleasantness and threat expectancy are also frequently used. Fear conditioning serves as a useful experimental model of exposure therapy, due to the use of ‘extinction’ and ‘return of fear’ phases (Zuj & Norrholm, 2019), Extinction can be conducted immediately after acquisition or after a delay of for instance 24 h. Return of fear can be assessed in different types of paradigms, such as renewal, where extinction is conducted in a context that differs from that of acquisition and the return of fear test. The differential responses (i.e., the difference in responding between the CS+ and CS−) can be measured to test how quickly the fear response to the CS+ is extinguished relative to a control, and subsequently, whether the extinguished fear response returns in response to a variety of manipulations aimed at modeling fear relapse (i.e., return of fear) after treatment (Lipp et al., 2020; Vervliet et al., 2013), such as after a break (modeling spontaneous recovery) or after the US is sporadically presented again following the CS+ (modeling re‐acquisition).

Despite increasing advocacy for methodological rigor in fear conditioning (Beckers et al., 2013; Lonsdorf, Klingelhöfer‐Jens, et al., 2019; Lonsdorf & Merz, 2017; Lonsdorf, Merz, & Fullana, 2019; Ney et al., 2018; Ney, Laing, et al., 2020), and psychological science more generally (Simmons et al., 2011), the experimental designs used in human conditioning research vary considerably using, for instance, different stimuli as CSs and USs. While this disparity has begun to be addressed in principle (Bach et al., 2022) and empirically (Ney, Laing, et al., 2020) there are still many research design options of unknown effects. One example is that fear conditioning paradigms often use different USs—most often an electric shock is used but loud tones (usually 100 dBA) and human screams are also routinely employed (Lipp, 2006; Lonsdorf et al., 2017). However, there is little comparative research assessing the effect of using different USs on conditioning outcomes. Understanding the difference in conditioning between USs is particularly important given that some fear conditioning research has begun to move online, where electric shocks (the most common US) cannot be administered (McGregor et al., 2021; Purves et al., 2019). Glenn et al. (2012) compared a human scream US to electric shock with fear‐potentiated startle as the physiological outcome measure and found that startle responses were significantly higher during the CSs paired with the shock relative to the scream, and the shock US was rated as more aversive. In contrast, Neumann and Waters (2006) reported that a loud, aversive scraping sound produced SCR and heart rate fear conditioning comparable to an electric shock and a loud tone. A shock, however, produced stronger fear conditioning (i.e., stronger acquisition and impaired extinction) compared to an air puff to the larynx and a foul odor (Busch & Evans, 1977; Murray & Carruthers, 1974). However, in a recent experiment that employed four times as many trials compared to an average fear conditioning paradigm, it was reported that a loud tone produced stronger fear conditioning compared to a shock in heart period, subjective ratings of CS valence/arousal, and skin conductance (Sperl et al., 2016), suggesting that the nature of the experimental design will affect how the US is responded to. A meta‐analysis also suggests that aversive film clips show equivalent conditioning when compared to electric shocks for fear‐potentiated startle, subjective ratings of US expectancy and CS pleasantness, but not for SCRs, where shock produces significantly stronger conditioning (Ney, Schenker, & Lipp, 2022).

One of the core principles of fear conditioning is that conditioning—whether it be fear acquisition or extinction—occurs when a participant's expectancy of what is about to occur is violated (Bouton et al., 2020; Craske et al., 2014; Rescorla & Wagner, 1972). Therefore, the premise of exposure therapy is that reduction in fear is due to continually presenting the patient with situations where their fear memory will be challenged and their expectancy of a bad outcome violated (Craske et al., 2014). Similarly, reducing the US reinforcement rate during acquisition to make it less predictable may impair extinction learning (Grady et al., 2016; Humphreys, 1940). However, to the best of our knowledge, no experiment has tested the effects of using multiple USs during acquisition that are presented in an unpredictable sequence on fear conditioning.

Fear conditioning is not only affected by parameters related to the conditioning procedure, but by individual differences as well (Lonsdorf & Merz, 2017). One potential factor that may influence fear conditioning is the endocannabinoid system (Morena et al., 2016; Ney, Akhurst, et al., 2021). The endocannabinoid system is a lipid signaling system that is present in both the peripheral and central nervous systems (Howlett et al., 2002; Ligresti et al., 2016). Arachidonoyl ethanolamide (AEA) and 2‐arachidonoyl glycerol (2‐AG) are able to interface with cannabinoid receptors and modulate memory and learning, including extinction (Devane et al., 1992; Kano et al., 2009; Ligresti et al., 2016; Suguira et al., 1995). Other n‐acyl ethanolamides such as oleoylethanolamide (OEA) do not interact directly with cannabinoid receptor 1 but are involved indirectly in endocannabinoid system function. Animal studies have systematically shown that impairment of endocannabinoid signaling (either via reduced endocannabinoid tone or receptor blockade) significantly reduces fear extinction whereas augmentation of endocannabinoid signaling improves extinction (Hill et al., 2018; Marsicano et al., 2002; Ney et al., 2019). An increasing number of human studies have found that AEA and 2‐AG concentration in blood is associated with fear extinction in the same direction identified in animal studies (Crombie et al., 2021; Mayo, Asratian, Lindé, Morena, et al., 2020; Ney, Crombie, et al., 2022; Ney, Matthews, et al., 2021; Spohrs et al., 2021), and genetic polymorphisms that are associated with increased AEA levels or receptor availability are correlated with improved fear extinction (Dincheva et al., 2015; Mayo, Asratian, Lindé, Holm, et al., 2020; Zabik et al., 2021). However, both genetics and blood biomarkers have notable drawbacks—genetic differences do not necessarily provide an indication of biological differences and blood samples are invasive and provide an acute measure that does not necessarily reflect an individual's typical biological profile. Hair sampling may resolve these disadvantages because hair samples provide a longitudinal (i.e., 1 cm of hair is approximately 1 month's analyte concentration) measure of biological profiling (Gao et al., 2022; Walther et al., 2023).

In the current study, we sought to answer two questions: Firstly, whether variability of the US during fear conditioning leads to exaggerated SCRs and whether endocannabinoid concentrations in hair are associated with fear conditioning. We hypothesized that variation of the US during acquisition would lead to lower habituation to the US, poorer extinction, and higher return of fear during renewal and reacquisition. We expected that the scream and shock USs would produce similar conditioning, based on the results of similar previous research (Neumann & Waters, 2006). Exploratory analyses were conducted on the relationship between hair endocannabinoids and fear conditioning outcomes. We hypothesized that AEA, 2‐AG, and OEA in hair samples would be significantly associated with fear conditioning, with higher AEA, 2‐AG, and OEA levels associated with faster extinction and lower return of fear during renewal and re‐acquisition. Unfortunately, due to technical analysis issues, data loss was experienced in the hair endocannabinoid analysis, such that these results are only interpretable as exploratory.

## 1 METHOD

### 1.1 Participants

One hundred ten participants were recruited into the current study from the Queensland University of Technology Psychology undergraduate cohort. Aspiring participants were excluded if they had a pacemaker, were taking anti‐cholinergic medications, had a heart condition, or were pregnant. Participants were otherwise not screened for other existing health issues. We were unsure of what effect size would be needed to measure hair endocannabinoid effects, since this was the first study testing this. Therefore, we based the sample size on our previous study (Lipp et al., 2021), which had a very similar design and was powered to detect a small to moderate difference in electrodermal indices between three groups (*f* = .18). Participants all provided informed consent and the study was approved by the university's Human Research Ethics Committee. Participants received either course credit or $25AUD after completing the study. One participant withdrew before completing the study, leaving 109 participants in the final sample. Participants were randomly allocated to receive either a Shock, a Scream, or 50% shocks and 50% screams (Shock/Scream) as the US during the experiment. There was *n* = 40 in the Shock group, *n* = 34 in the Scream group and *n* = 35 in the Shock/Scream group.

### 1.2 Materials and measures

#### 1.2.1 Survey questionnaires

We used a number of different pen‐and‐paper anxiety and depression symptomology questionnaires during the current study to test for group‐level differences. The Intolerance of Uncertainty Scale (IUS‐12; Carleton et al., 2007) measures feelings and responses to ambiguity and uncertainty about the future. The Anxious Arousal (AA) and Anhedonic Depression (AD) items from the Mood and Anxiety Symptom Questionnaire (MASQ; Clark & Watson, 1991; Watson et al., 1995) measure anxious arousal and depressive symptoms using 39 items. The Mood and Feelings Questionnaire (MFQ; Angold et al., 1995) was used to measure recent depressive symptomology (over the past 2 weeks). The Penn State Worry Questionnaire (PSWQ; Meyer et al., 1990) is an index of trait worrying and the State‐Trait Anxiety Inventory (STAI; Spielberger et al., 1983) is a measure of both trait and current anxiety. The questionnaires were used in this study to ensure that participant groups did not differ significantly in personality, mood, and anxiety that might impact on the study outcomes.

#### 1.2.2 Stimuli

The CS+ and CS− were counterbalanced from a pool of three animal photos (a bird, a fish, and a frog). CSs were presented on a computer screen for 6 s each, with an intertrial interval of between 14 and 18 s (average = 16 s). During acquisition and re‐acquisition, the CS+ was followed by a US that coincided with CS+ offset. The reinforcement rate was 100% during acquisition and 50% during re‐acquisition. The sequence of CS+ and CS− was pseudorandom such that they were never presented more than two times in a row. The shock US was generated using a Digitimer DS7A stimulator unit and consisted of a sequence of three 2 ms electro‐tactile stimuli presented 16 ms apart (perceived as one stimulus). A shock work‐up procedure was used to calibrate the shock stimulus individually to a level that was perceived as ‘unpleasant, but not painful’. This is described in the Procedure. The scream US was a 6 s screaming lady recording standardly used in fear conditioning (International Affective Digitized Sounds, sound number 277; Bradley and Lang [2000, 2007]) and was presented at 90 dBA. The background color of the computer screen changed between phases to implement an ABA renewal design. Color A was present continuously during habituation, acquisition, renewal, and re‐acquisition, whereas color B was present only during extinction. Background colors were counterbalanced between participants and were either pink (RBG 255128192) or blue (RBG 0128255). The background color onset preceded the first CS by 5 s. The conditioning task was programmed using DMDX (Forster & Forster, 2003).

#### 1.2.3 Physiological recordings

Skin conductance and respiration were recorded during the current study. Pre‐gelled self‐adhesive electrodes (BIOPAC EL507) were attached to the thenar and hypothenar eminences of the non‐dominant hand to record skin conductance through an MP150 BIOPAC system coupled with an EDA100C amplifier. Respiration was collected as a control measure when SCRs were scored and was recorded with an RSP1000C amplifier by fitting a single‐belt transducer (BIOPAC TSD201) around the torso of each participant. Respiration and skin conductance was recorded at a sampling frequency of 1000 Hz using Acqknowledge v3.9.1.

#### 1.2.4 Subjective ratings

Subjective anxiety, CS pleasantness, and CS arousal ratings were collected during the experiment at baseline (prior to habituation), after acquisition, after extinction, after renewal test, and after re‐acquisition. To assess subjective anxiety, participants were asked to indicate their response on a Likert scale ranging from 1 (not at all anxious) to 9 (very anxious). To assess CS pleasantness, participants saw a picture of the relevant CS and were asked to rate how pleasant they found the image on a scale from 1 (very unpleasant) to 9 (very pleasant), with 5 being ‘neutral’. To assess CS arousal, participants were asked to rate how arousing they found the images on a scale from 1 (very calming) to 9 (very exciting), with 5 being ‘neutral’. Ratings were completed using their dominant hand on a keyboard and the ratings task was programmed and the data recorded using DMDX. Contingency awareness was assessed after acquisition by asking: ‘Did you notice a relationship between the animal pictures and the electrical stimulus? 1. The electrical stimulus was always preceded by the bird, 2. The electrical stimulus was always preceded by the fish, 3. The electrical stimulus was always preceded by the frog, 4. There was no relationship between the animal pictures and the electrical stimulus, 5. I could not tell.’ In total, 19 of the final 109 participants (17.4%) reported not having contingency awareness and there were no significant differences between the groups for this measure. All statistical analyses were repeated with and without aware participants, and no differences in the main results—except for one result in a third interval response during the renewal phase—were observed. This discrepancy is reported in the results.

#### 1.2.5 Procedure

**FIGURE 1:**
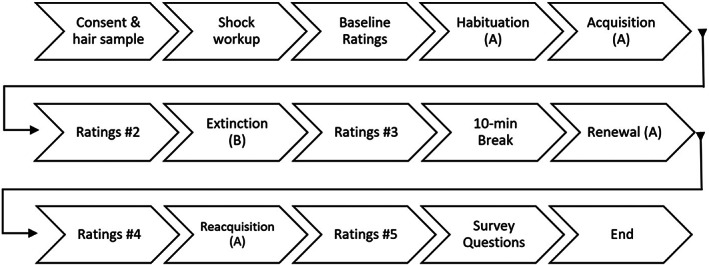
Experimental design. Habituation, acquisition, renewal and reacquisition are in Context A and extinction is in Context B.

The procedure for the experiment is visualized in Figure 1. When arriving at the laboratory, participants washed their hands and the experimenter took a hair sample, with 3 cm collected from the scalp using scissors. Samples were stored at room temperature until analysis and were analyzed within 18 months of collection. Next, participants had the skin conductance electrodes, shock electrode, and respiration transducer belt attached. They were told that they would receive electric shocks and hear a loud unpleasant noise through headphones and would be required to watch a series of images of animals and fill out mood and survey questions. All participants received the same instructions so that instruction‐based anticipation was consistent across groups. Before starting the experiment, all participants except for those in the Scream group completed a shock work‐up procedure, where the experimenter presented a series of electric shocks of increasing intensity (1 mA intervals, beginning at 0 mA). When the shock intensity was reported to be ‘unpleasant, but not painful’, this was set as the level that would be used throughout the experiment. Next, a 3‐minute baseline recording of physiology was performed. Following this, participants completed the baseline (pre‐habituation) ratings task, where they entered rating data for anxiety, CS pleasantness, and CS arousal.

Participants then began the habituation phase. During habituation, participants were presented with 4 CS+ and 4 CS− trials in context A (context A was either a blue or pink background present throughout the phase and counterbalanced across participants—see Stimuli). Immediately following habituation, participants underwent acquisition, where they either received 8 shocks, 8 screams, or 4 shocks and 4 screams following the 8 CS+ trials. 8 CS− trials were also presented and these were not followed by the US. The background during acquisition remained as context A and was present throughout the phase. The CS+/CS− order was counterbalanced so that half of the participants saw CS+ and half saw CS− on the first acquisition trial, and pseudorandomised such that CS+ or CS− were never presented more than two times in succession. Presentation of the scream and shock USs in the Shock/Scream condition were pseudorandom, such that neither shock nor scream were presented more than twice in succession. There were two orders of USs in this condition that were counterbalanced across participants: Shock, shock, scream, shock, scream, scream, shock, scream, and the inverse of this sequence. At the end of acquisition, participants completed the second ratings task on the computer. After this, participants underwent the extinction phase, where 24 trials of each CS were presented in context B without reinforcement. After this, participants completed the third ratings task and then had a 10‐minute break, where they read a section of a university textbook. After this break, participants completed the renewal phase, which consisted of four unreinforced CS+ and four unreinforced CS− presentations in context A. After this, participants completed the fourth ratings task before completing the final conditioning phase (re‐acquisition). During re‐acquisition, participants viewed eight CS+ and CS− presentations, with 50% of the CS + s followed by either a shock (Shock group), a scream (Scream group) or either a shock or a scream (two presentations each, Shock/Scream group). After re‐acquisition, participants completed the final ratings task and removed the physiological equipment and shock electrode. They then completed the survey questionnaires before being thanked for their time, reimbursed, and debriefed on the purposes of the experiment.

#### 1.2.6 Data pre‐processing and analyses

##### 1.2.6.1 Hair analysis

Hair samples were weighed and washed for 3 minutes with 2 mL isopropanol before being incubated in 1.6 mL methanol overnight. A 50 μL surrogate standard mixture containing d5‐AG, d4‐AEA, d4‐cortisol, ^13^C_3_‐progesterone, and d4‐OEA was added prior to incubation to assist with quantification of the native analytes. The samples were evaporated to dryness under nitrogen twice the following day and re‐constituted in 150 μL acetonitrile and then 30 μL chloroform as the final solution. 2 μL of the sample was injected into a Waters Acquity® H‐class UPLC (Waters Corporation, Milford, MA), equipped with a Waters Acquity BEH C18 column and paired with a Waters Xevo® TQ triple quadrupole mass spectrometer (Waters Corporation). Full details of the analytical method and mass spectrometry conditions are described in Ney, Felmingham, et al. (2021) with the mass transitions and chromatographic separation described in Ney, Felmingham, et al. (2020). Hair levels of each respective analyte were correlated against the outcomes throughout the fear conditioning experiment by including them as a continuous covariate variable in mixed ANOVA models.

Due to the primary researcher relocating universities during the project (LJN), there was a considerable time in between hair sample collection and analysis. This was further complicated by mass spectrometry instrument technical issues that required repairs and during analysis, the instrument showed very low sensitivity compared to our previous work. Therefore, AEA and 2‐AG were quantifiable in just over 30 hair samples each, and cortisol and progesterone were not quantifiable in the majority of samples. Analyses for AEA (*n* = 31: 12 in Shock, 8 in Scream, and 11 in Shock/Scream) and 2‐AG (*n* = 34: 10 in Shock, 11 in Scream, and 13 in Shock/Scream) are conducted including only participants with values above the limit of quantification (Ney, Felmingham, et al., 2021). OEA was quantifiable in all samples. Due to these limitations, all endocannabinoid analyses are treated as exploratory in the present study.

##### 1.2.6.2 Ratings data

Participant anxiety as well as CS pleasantness and arousal ratings were analyzed using 3 (Group: Shock, Scream, Shock/Scream) × 5 (Phase: Baseline, Post‐Acquisition, Post‐Extinction, Post‐Renewal, Post‐Reacquisition) × 2 (CS: CS+, CS−) mixed ANOVAs, though analysis of the anxiety ratings did not include the CS factor.

##### 1.2.6.3 Skin conductance responses

SCR data were scored manually by three researchers (L.N. and two research assistants) using the trough‐to‐peak method with the Acqknowledge 3.9.1 interface. SCRs were quantified separately for each of three latency intervals (Prokasy & Ebel, 1967). The first interval response (FIR) was scored as an SCR beginning within 1–4 s from CS onset, whereas the second interval response (SIR) and third interval response (TIR) were scored as SCRs beginning within 4–7 and 7–10 s from CS onset, respectively. Scoring SCRs in separate intervals aims to exploit the information available in a skin conductance trace during a long‐lasting CS in more detail as, although not independent, each component can provide information on different psychological processes (Luck & Lipp, 2016). Whereas FIRs are believed to index orienting to CS onset, SIRs and TIRs are thought to index US anticipation and reactivity to the presence (or absence) of the US, respectively (Prokasy et al., 1973). SCRs were square root transformed and range corrected prior to analysis. Blocks were created by averaging the SCRs of two sequential trials of each CS to account for the high variability of SCRs across single trials (Lonsdorf et al., 2017; Ney, Luck, et al., 2022).

3 (Group: Shock, Scream, Shock/Scream) × *n* (Block: average of two trial trials) × 2 (CS: CS+, CS−) mixed ANOVAs using the SCR data were performed separately for each phase. There were 2 blocks in habituation and renewal, 4 blocks in acquisition and re‐acquisition, and 12 blocks in extinction. Post hoc pairwise comparisons were performed using the Least Significant Difference correction. Due to the repeated measures design, we tested for sphericity in the data by using Mauchly's test. In the event that Mauchly's test was significant, Greenhouse Geisser (GG) corrections were applied, and adjusted degrees of freedom were reported. All data analyses were performed using SPSS v27. Bayesian analyses were conducted using the *jsp* plugin in Jamovi 2.25 for all analyses involving Group factors and are reported as Bayes Factors (BF_10_), where 0.01–0.03 is very strong evidence for the null hypothesis, 0.10–0.33 is substantial evidence for the null hypothesis, 1 is no evidence, 1–3 is anecdotal evidence for the alternative hypothesis, 3–10 is substantial evidence for the alternative hypothesis, and higher as increasing strong evidence for the alternative hypothesis.

## 2 RESULTS

### 2.1 Demographics

There were no significant differences between groups in age or gender distribution. The groups also did not differ on any of the self‐report questionnaires or shock intensity (Table 1). Four participants did not report the questionnaires in full, and these responses were removed from the analyses conducted below.

**TABLE 1:** Demographics and questionnaire data for the Shock, Scream, and Shock/Scream groups.

	Shock	Scream	Shock/scream	Difference
Age (*SD*)	19.95 (2.84)	19.85 (1.97)	21.00 (6.12)	*F*(2,105) = 0.88, *p* = .419
Gender (M/F)	7/33	12/22	9/26	*χ* ^*2*^(2) = 3.05, *p* = .218
IUS‐12	34.21 (10.75)	29.91 (8.42)	33.74 (9.39)	*F*(2,106) = 2.02, *p* = .138
MASQ‐AA	31.15 (9.68)	30.76 (10.59)	30.20 (10.35)	*F*(2,106) = 0.08, *p* = .922
MASQ‐AD	61.93 (15.92)	58.53 (18.19)	62.94 (16.73(	*F*(2,106) = 0.65, *p* = .526
MFQ	7.88 (4.66)	7.29 (5.31)	8.91 (4.22)	*F*(2,106) = 1.04, *p* = .358
PSWQ	56.54 (14.49)	52.22 (14.32)	58.71 (13.54)	*F*(2,103) = 1.82, *p* = .167
STAI‐S	39.58 (8.77)	43.26 (12.35)	42.23 (7.20)	*F*(2,106) = 1.47, *p* = .167
STAI‐T	46.08 (11.27)	43.62 (11.31)	47.77 (10.56)	*F*(2,106) = 1.23, *p* = .297
Shock intensity	9.8 (8.4)		7.9 (6.8)	*t*(73) = 1.02, *p* = .312

### 2.2 Electrodermal responding

Due to poor quality skin conductance data, non‐responding and experimenter error, some participants' skin conductance responses (SCRs) were missing from the habituation (*n* = 3: Shock = 2, Scream = 1), acquisition (*n* = 3: Shock = 2, Shock/Scream = 1), extinction (*n* = 2: Shock = 1, Shock/Scream = 1), and re‐acquisition (*n* = 3: Shock = 2, Scream = 1) phases.

### 2.3 Electrodermal first interval responses

**FIGURE 2:**
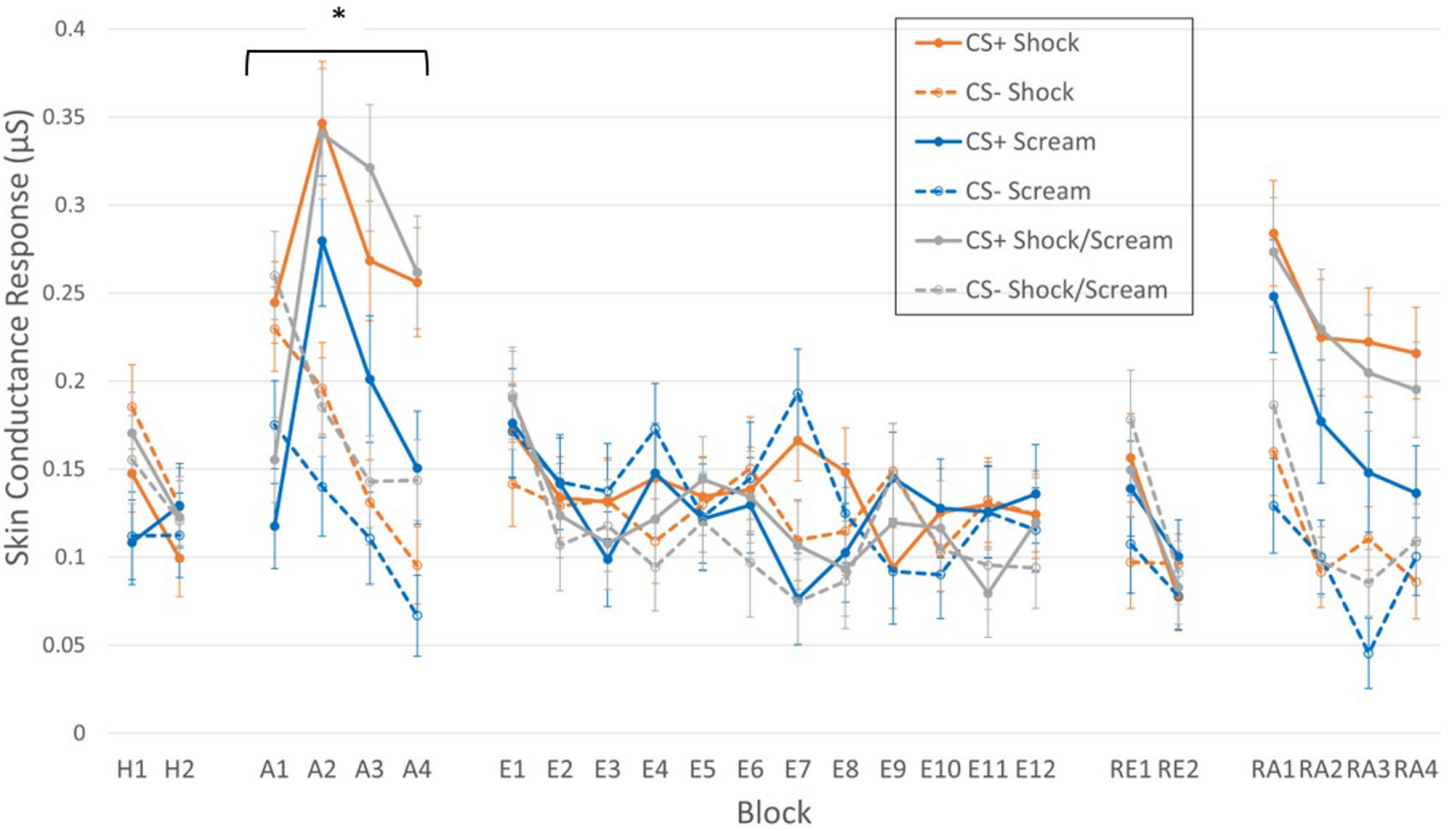
First Interval Responses throughout the experiment. A, acquisition; E, extinction; H, habituation; RA, reacquisition; RE, renewal. During acquisition, significantly higher responses were observed in the Shock and Shock/Scream groups compared to the Scream group. *Group‐level effect *p* < .05.

Figure 2 visualizes the FIR SCRs throughout the experiment. During habituation, there was a significant effect of Block, *F*(1,103) = 13.16, *p* < .001, ${\eta_{p}}^{2}$ = .11, with responses reducing across trials. There was a near‐significant Block × Group interaction, *F*(2,206) = 3.06, *p* = .051, ${\eta_{p}}^{2}$ = .06, with the Scream group showing lower overall responding in the first half of habituation compared to Shock (*p* = .016, BF_10_ = 0.58) and Shock/Scream (*p* = .046, BF_10_ = 0.37) groups, though responding was equivalent between these groups in the second half of habituation (BF_10_ = 0.01). All other effects were non‐significant, *F* < 2, smallest *p* = .169, largest ${\eta_{p}}^{2}$ = .03.

During acquisition, there were significant main effects of CS, *F*(1,103) = 56.04, = *p* < .001, ${\eta_{p}}^{2}$ = .35 and Block, GG‐corrected *F*(3,273) = 14.35, *p* < .001, ${\eta_{p}}^{2}$ = .12. There was also a significant effect of Group, *F*(2,103) = 4.32, *p* = .016, ${\eta_{p}}^{2}$ = .08, with overall SCR responses larger in the Shock and Shock/Scream groups compared to the Scream group (*p* = .014, BF_10_ = 4.29 and *p* = .010, BF_10_ = 4.56 respectively), but Shock and Shock/Scream groups did not differ significantly (*p* = .841, BF_10_ = 0.25). There was a significant CS × Block effect, *F*(3,309) = 28.54, *p* < .001, ${\eta_{p}}^{2}$ = .22, with the difference between CS+ and CS− SCRs larger in Blocks 2–4 (mean difference: 0.121 to 0.148, *p* < .001) compared to Block 1 (mean difference: 0.049, *p* = .006). There were no other significant effects, *F* < 1.7, smallest *p* = .137, largest ${\eta_{p}}^{2}$ = .03.

During extinction, there was a significant main effect of Block, GG‐corrected *F*(9, 929) = 3.46, *p* < .001, ${\eta_{p}}^{2}$ = .03. There was also a CS × Block × Group interaction, GG‐corrected *F*(18,943) = 1.66, *p* = .041, ${\eta_{p}}^{2}$ = .03, which was driven by a larger SCR to CS− compared to CS+ in the Scream group on Block 7 (*p* < .001, BF_10_ = 313.41), whereas the Shock group had slightly larger SCRs to CS+ compared to CS− during Block 7 (*p* = .036, BF_10_ = 1.05). There were no extreme outlying values in the data and no experimental manipulations occurring during Block 7, so this effect is difficult to explain other than random chance. All other effects were non‐significant, *F* < 1.2, smallest *p* = .329, largest ${\eta_{p}}^{2}$ = .02.

During renewal, there was a significant effect of Block, *F*(1,106) = 19.21, *p* < .001, ${\eta_{p}}^{2}$ = .15, with SCRs reducing across blocks. No other effects were significant during renewal, *F* < 2.3, smallest *p* = .107, largest ${\eta_{p}}^{2}$ = .04; however, on the first Block there was a significant difference between CS+ and CS− in the Shock condition (*p* = .046) but in no other condition (*ps* > .3). During re‐acquisition, there were significant CS, *F*(1,103) = 60.35, *p* < .001, ${\eta_{p}}^{2}$ = .37 and Block, *F*(3,309) = 20.80, *p* < .001, ${\eta_{p}}^{2}$ = .17, main effects, with larger responses to the CS+ and responses reducing across blocks. No other effects were significant during re‐acquisition, *F* < 1, smallest *p* = .465, largest ${\eta_{p}}^{2}$ = .02.

### 2.4 Electrodermal second interval responses

**FIGURE 3:**
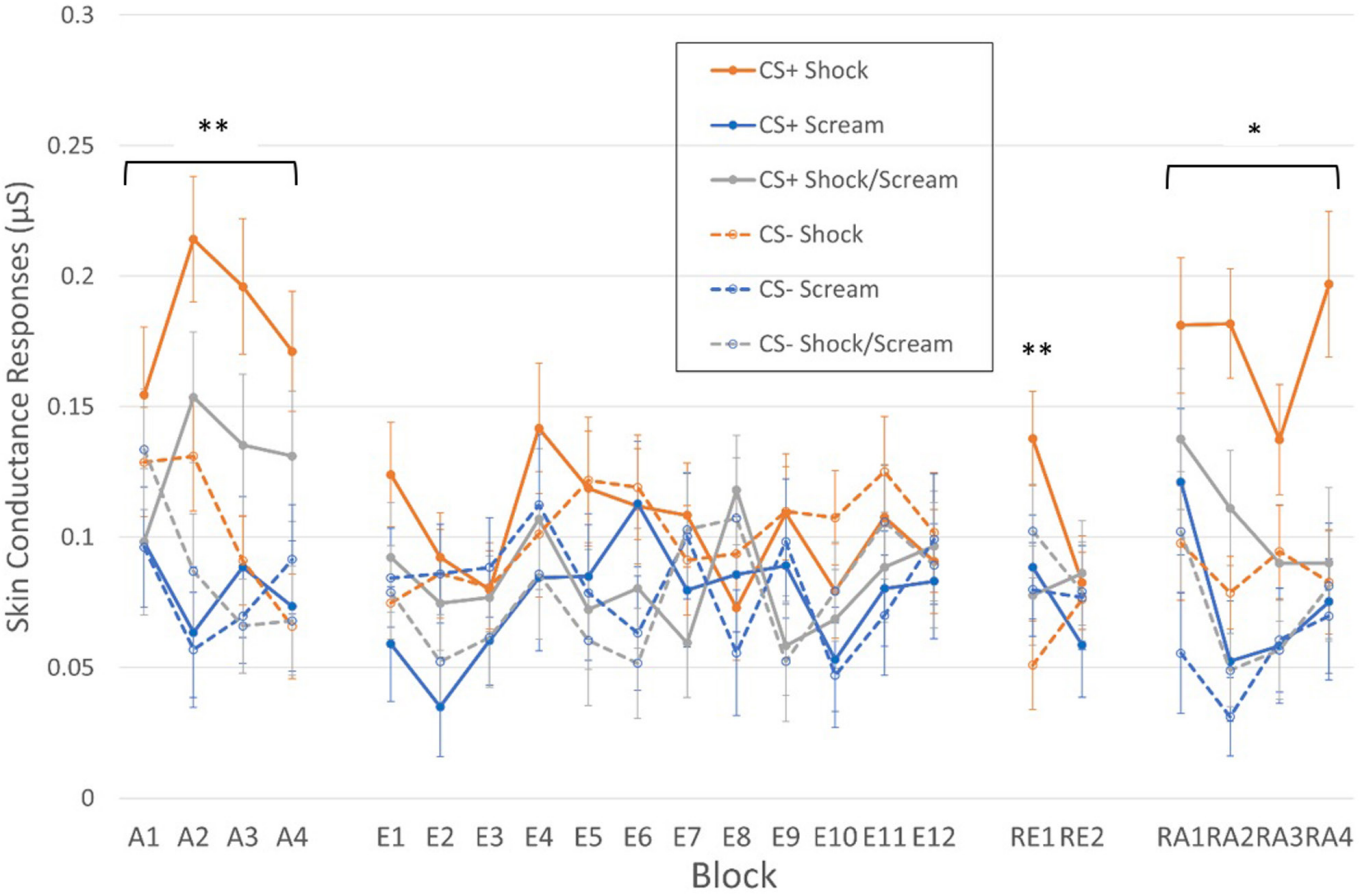
Second Interval Responses throughout the experiment. A, acquisition; E, extinction; H, habituation; RA, reacquisition; RE, renewal. CS+/CS− differential responding was observed in the Shock and Shock/Scream groups, but not the Scream group, during acquisition and reacquisition. Only the Shock group displayed differential renewal. ** Group‐level effect significant at *p* < .01. *Group‐level effect significant at *p* < .05.

Electrodermal SIRs are depicted between groups and CSs and across phases in Figure 3. During acquisition, there were significant main effects of CS, *F*(1,103) = 17.79, *p* < .001, ${\eta_{p}}^{2}$ = .15 and Group, *F*(2,103) = 6.61, *p* = .002, ${\eta_{p}}^{2}$ = .11. There were also significant CS × Group, *F*(2,103) = 5.39, *p* = .006, ${\eta_{p}}^{2}$ = .10, and CS × Block interactions, GG‐corrected *F*(3,295) = 3.02, *p* = .032, ${\eta_{p}}^{2}$ = .03. The CS × Group interaction was due to differential responding in the Shock (mean difference SCR: 0.08, *p* < .001, BF_10_ = 195.00) and Shock/Scream (mean difference SCR: .041, *p* = .019, BF_10_ = 2.37) groups but not in the Scream group (mean difference SCR: .002, *p* = .891, BF_10_ = 0.19). This effect was driven by larger responses to CS+ in the two groups displaying differential responses, with no differences in responses to CS−. The CS × Block interaction was again driven by an absence of differential responding during Block 1 (*p* = .881), whereas differential responding was present in Blocks 2–4 (*p* = .003, *p* < .001, *p* = .006, respectively). No other significant differences were found, *F* < 2, smallest *p* = .164, largest ${\eta_{p}}^{2}$ = .03.

During extinction, all effects were non‐significant, largest effect main effect for Block, GG‐corrected *F*(9,932) = 1.72, *p* = .080, ${\eta_{p}}^{2}$ = .02.

During renewal, there was a significant CS × Block × Group interaction, *F*(2,106) = 5.17, *p* = .007, ${\eta_{p}}^{2}$ = .09. These interactions were driven by only the Shock group having differential SCRs during the first (*p* < .001, BF_10_ = 93.70) but not the second (*p* = .746, BF_10_ = 0.18) block of renewal, whereas the other groups did not have differential SCRs (smallest *p* = .315, BF_10_ = 0.31). No other effects were significant, largest *F* = 3.04, smallest *p* = .084, largest ${\eta_{p}}^{2}$ = .03.

During re‐acquisition, there were significant main effects of CS, *F*(1,103) = 21.41, *p* < .001, ${\eta_{p}}^{2}$ = .17, Block, GG‐corrected *F*(3,273) = 3.72, *p* = .016, ${\eta_{p}}^{2}$ = .04, and Group, *F*(2,103) = 7.63, *p* < .001, ${\eta_{p}}^{2}$ = .13. The CS and Group effects were qualified by a significant CS × Group interaction, *F*(2,103) = 3.64, *p* = .030, ${\eta_{p}}^{2}$ = .07, where differential SCRs were displayed by the Shock group (mean difference SCR: 0.086, *p* < .001, BF_10_ = 166) and to a lesser extent the Shock/Scream group (mean difference SCR: 0.035, *p* = .055, BF_10_ = 1.13) but not the Scream group (mean difference SCR: 0.023, *p* = .225, BF_10_ = 0.55). There were no other significant differences in the analysis, *F* < 2, smallest *p* = .256, largest ${\eta_{p}}^{2}$ = .01.

### 2.5 Electrodermal third interval responses

**FIGURE 4:**
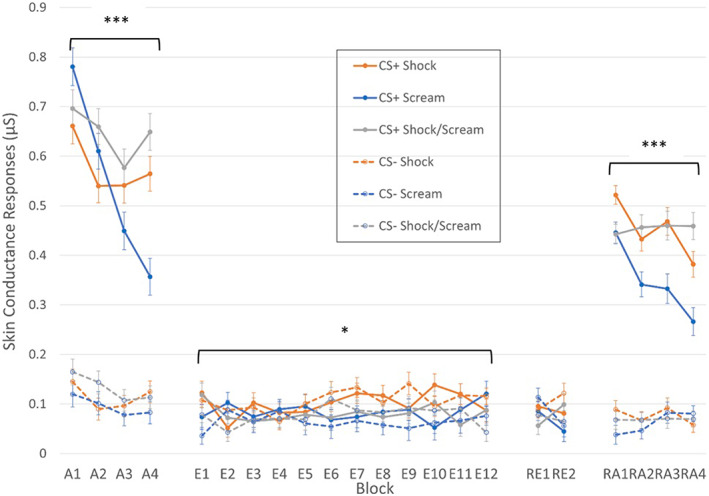
Third Interval Responses throughout the experiment. A, acquisition; E, extinction; H, habituation; RA, reacquisition; RE, renewal. Faster habituation to the US was observed during acquisition in the Scream group, but stronger differential responding in the Scream group was observed during extinction. Higher responses to the CS+ in the Shock compared to Scream group were observed during reacquisition. ***Group‐level effect significant at *p* < .001. *Group‐level effect significant at *p* < .05.

Figure 4 shows the TIR data throughout the experiment. During acquisition, there were significant main effects of CS, *F*(1,103) = 47.98, *p* < .001, ${\eta_{p}}^{2}$ = .86, Block, *F*(3,266) = 32.00, *p* < .001, ${\eta_{p}}^{2}$ = .24, and Group, *F*(2,103) = 4.25, *p* = .017, ${\eta_{p}}^{2}$ = .08. There were also Block × Group, *F*(5,266) = 7.76, *p* < .001, ${\eta_{p}}^{2}$ = .13 and CS × Block interactions, *F*(3,261) = 14.23, *p* < .001, ${\eta_{p}}^{2}$ = .12, which were qualified by a significant CS × Block × Group interaction, *F*(5,261) = 7.43, *p* < .001, ${\eta_{p}}^{2}$ = .13. This three‐way interaction was the result of initially high but continuously reducing SCRs following the CS+ in the Scream group compared to slower habituation to the US in the other groups, and slower habituation to the US in the Shock/Scream group compared to the Shock group (mean difference SCR in Block 2: 0.120, *p* = .017). The CS × Group interaction was not significant, *F*(2,103) = 0.90, *p* = .410, ${\eta_{p}}^{2}$ = .02.

During extinction, there was a significant CS × Group interaction, *F*(2,104) = 3.29, *p* = .041, ${\eta_{p}}^{2}$ = .06, which was due to larger SCRs to the CS+ compared to CS− in the Scream (*p* = .007, BF_10_ = 1.84), but not the Shock (*p* = .447, BF_10_ = 0.22) or Shock/Scream (*p* = .633, BF_10_ = 0.19) groups. All other effects were not significant, highest *F* = 2.31, smallest *p* = .109, largest ${\eta_{p}}^{2}$ = .03.

During renewal, there were no significant effects, largest *F* = 2.91, smallest *p* = .059, largest *η*_*p*_^*2*^ = .04.

For re‐acquisition, there were significant main effects of CS, *F*(1,103) = 694.81, *p* < .001, ${\eta_{p}}^{2}$ = .87, Block, GG‐corrected *F*(3,289) = 8.04, *p* < .001, ${\eta_{p}}^{2}$ = .07, and Group, *F*(2,103) = 8.88, *p* < .001, ${\eta_{p}}^{2}$ = .15. There were also significant CS × Group, *F*(2,103) = 5.66, *p* = .005, ${\eta_{p}}^{2}$ = .10, Block × Group, *F*(6,309) = 2.93, *p* = .009, ${\eta_{p}}^{2}$ = .05, CS × Block, *F*(3,309) = 10.06, *p* < .001, ${\eta_{p}}^{2}$ = .09, and CS × Block × Group interactions, *F*(6,309) = 4.39, *p* < .001, *η*_*p*_^*2*^ = .08. The three‐way interaction was explained by larger SCRs after CS+ in the Shock compared to Scream group across all four blocks (*p* = .008, BF_10_ = 5.71, *p* = .009, BF_10_ = 4.53, *p* = .001, BF_10_ = 38.47, *p* = .003, BF_10_ = 8.81 respectively), but higher SCRs after the CS+ only in Block 1 (*p* = .005, BF_10_ = 8.92) and otherwise lower SCRs (or equal in the case of Block 3) to the CS+ (*p* = .043, BF_10_ = 0.25) compared to the Shock/Scream group. There were no significant differences between the groups across re‐acquisition blocks in response after the CS−.

### 2.6 Subjective ratings

A number of participants either missed items or reported erroneous values during the subjective ratings (*n* = 19: Shock = 8, Scream = 7, Shock/Scream = 4). These responses were not included in the analyses. The group breakdown for the 19 participants who did not exhibit contingency awareness was as follows: Shock = 6, Scream = 4, Shock/Scream = 9. The difference in contingency awareness between groups was not significant: *χ* ^*2*^(df = 2, *N* = 109) = 2.59, *p* = .274.

### 2.7 CS pleasantness ratings

**FIGURE 5:**
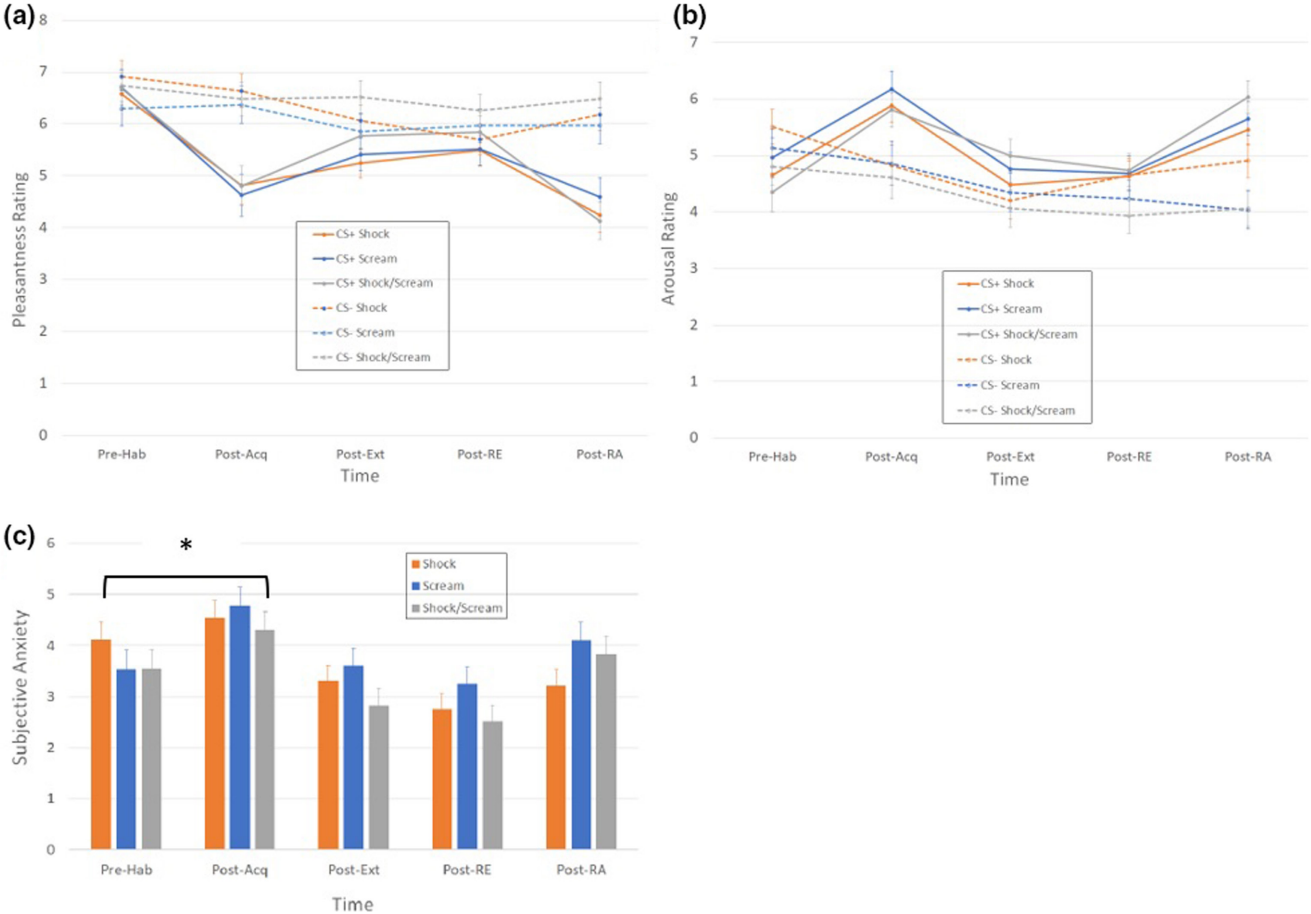
Subjective ratings of CS Pleasantness (Panel a), CS Arousal (Panel b), and overall Anxiety throughout the experiment. Acq, acquisition; Ext, extinction; Hab, habituation; RA, reacquisition; RE, renewal. Higher Anxiety was reported by the Scream group compared to the Shock and Shock/Scream after acquisition, but no other Group effects were observed in subjective ratings throughout the experiment. *Group‐level effect significant at *p* < .05.

Figure 5a displays the CS pleasantness ratings for the CS+ and CS− between groups and across phases. There were significant CS, *F*(1,88) = 21.55, *p* < .001, ${\eta_{p}}^{2}$ = .20 and Block, GG‐corrected *F*(3,286) = 26.10, *p* < .001, ${\eta_{p}}^{2}$ = .23 main effects, which were qualified by a significant CS × Block interaction, GG‐corrected *F*(7,286) = 25.00, *p* < .001, ${\eta_{p}}^{2}$ = .22. This interaction reflects a significant decrease in rated CS+ pleasantness from pre‐ to post‐acquisition (*p* < .001), which was accompanied by a less substantive but still significant decrease in rated CS− pleasantness after extinction (*p* < .01). In comparison, there were no significant differences between CS+ and CS− ratings prior to habituation. There were no other significant effects, *F* < 1, smallest *p* = .576, largest ${\eta_{p}}^{2}$ = .02.

### 2.8 CS arousal ratings

Figure 5b illustrates the arousal ratings for the CS+ and the CS− between groups and across blocks. Similar to pleasantness ratings, there were significant CS, *F*(1,92) = 14.27, *p* < .001, ${\eta_{p}}^{2}$ = .13, Block, GG‐corrected *F*(3,277) = 12.99, *p* < .001, ${\eta_{p}}^{2}$ = .12, and CS × Block effects, GG‐corrected *F*(3,283) = 18.73, *p* < .001, ${\eta_{p}}^{2}$ = .17. The interaction effect reflected larger differences in rated arousal between CS+ and CS− after acquisition and re‐acquisition (mean difference: 1.187 and 1.377, respectively, both *p* < .001) than during baseline (mean difference: −0.494, *p* = .015 – CS− rated as more arousing prior to habituation), extinction (mean difference: 0.545, *p* = .003), and renewal (mean difference: 0.409, *p* = .027). All other effects were non‐significant, *F* < 2, smallest *p* = .174, largest ${\eta_{p}}^{2}$ = .04.

### 2.9 Subjective anxiety

Figure 5c illustrates the anxiety ratings of participants across the experiment. There was a significant effect of Phase, *F*(3,257) = 32.35, *p* < .001, ${\eta_{p}}^{2}$ = .27, which was qualified by a significant Phase × Group effect, GG‐corrected *F*(6,257) = 2.81, *p* = .012, ${\eta_{p}}^{2}$ = .06, but the main effect of Group was not significant, *F*(2,87) = 0.57, *p* = .567, ${\eta_{p}}^{2}$ = .01. The interaction effect reflected significantly higher increases in anxiety in the Scream group during acquisition and re‐acquisition (relative to habituation levels) compared to the other groups. This is likely due to the absence of a shock‐work up procedure in the Scream group.

### 2.10 Endocannabinoid analyses and relation to fear conditioning

Exploratory analyses were conducted between endocannabinoid hair levels and fear conditioning using mixed model ANOVAs. Note that, due to the small sample sizes (due to data loss), the groups were collapsed for these analyses. There were no significant effects of AEA, 2‐AG, or OEA during habituation (all *ps* > .05). For FIR, there were no significant effects of AEA during acquisition, renewal, or reacquisition (*ps* > .05). However, there was a main effect of AEA during extinction, *F*(1,29) = 9.30, *p* = .005, ${\eta_{p}}^{2}$ = .24, as well as a Block × AEA interaction during extinction, *F*(11,319) = 2.65, *p* = .003, ${\eta_{p}}^{2}$ = .08. In extinction, higher hair AEA was associated with higher FIRs, except in the first Block where higher hair AEA was associated with lower FIRs. There was a significant Block × 2‐AG effect during extinction, *F*(11,352) = 2.08, *p* = .021, ${\eta_{p}}^{2}$ = .06, but no other effects during any other phase (all *ps* > .05). This significant interaction was in the opposite direction to AEA, such that higher hair 2‐AG was associated with lower FIRs across all extinction Blocks except for the first. There were no significant effects of OEA during acquisition, extinction, renewal, or re‐acquisition (all *ps* > .05).

For SIR, there were no significant effects of AEA during acquisition, renewal, or re‐acquisition (all *ps* > .05). There was a significant main effect during extinction, *F*(1,29) = 10.83, *p* = .003, ${\eta_{p}}^{2}$ = .27. These effects again suggested that higher hair AEA was associated with higher SIRs during extinction. There were no significant effects of 2‐AG for SIRs throughout the experiment (all *ps* > .05). There were no significant effects of OEA during acquisition, extinction, or re‐acquisition (all *ps* > .05). However, there was a significant main effect of OEA during renewal, *F*(1,91) = 7.45, *p* = .008, ${\eta_{p}}^{2}$ = .08, where higher OEA levels were associated with lower SIRs.

**FIGURE 6:**
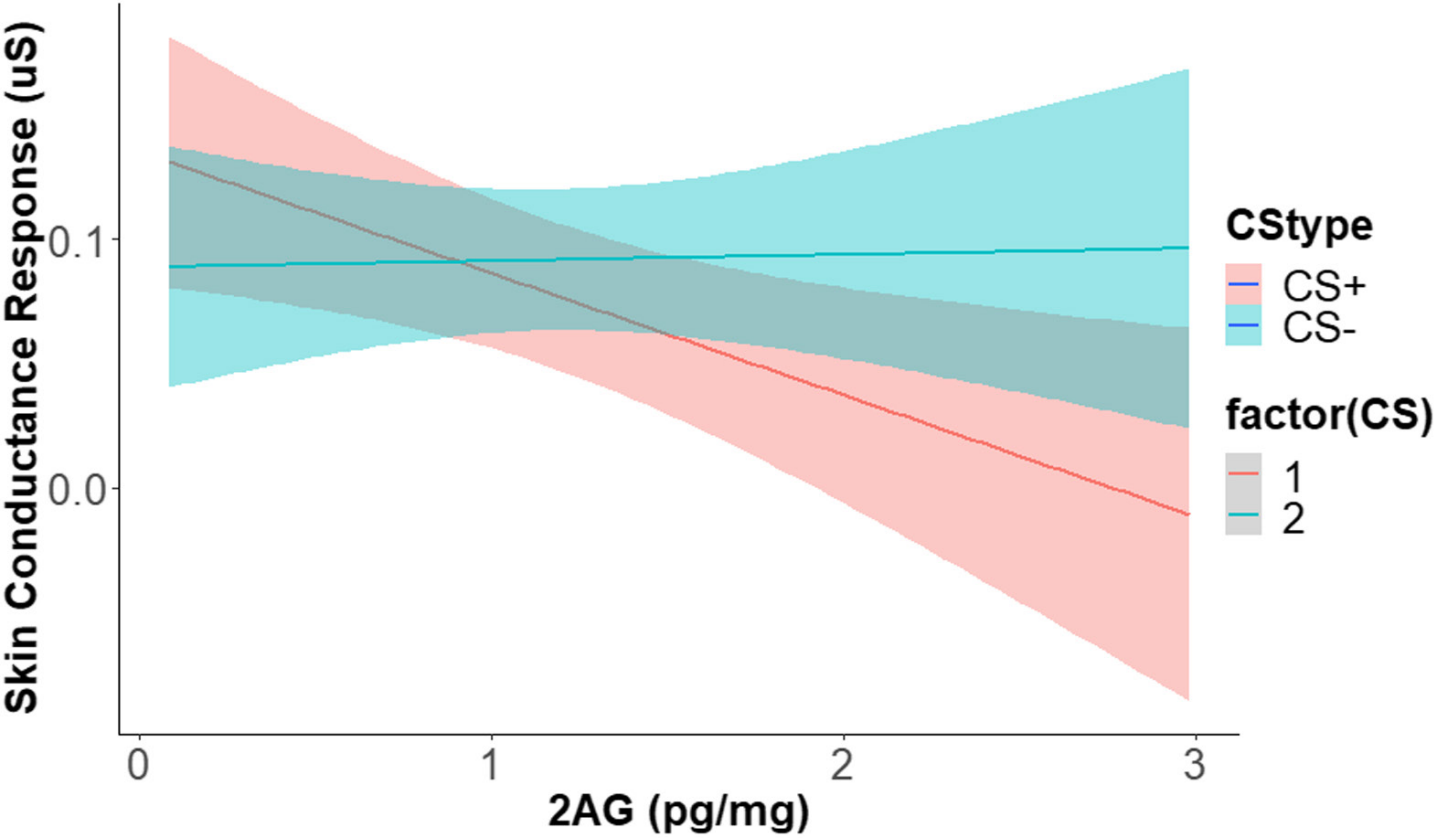
Higher 2‐arachidonoyl glycerol (2‐AG) hair concentrations were associated with lower differential fear renewal of third interval responses. This data is preliminary due to the small sample size.

For TIR there were no significant effects of AEA during acquisition, extinction, renewal, or re‐acquisition (all *ps* > .05). There were no significant effects of 2‐AG during acquisition, extinction, or re‐acquisition (all *ps* > .05), but there was a CS × 2‐AG interaction during renewal, *F*(1,32) = 4.71, *p* = .037, ${\eta_{p}}^{2}$ = .13. Lower TIRs after the CS+ during renewal were associated with higher 2‐AG, but there was no difference for the CS− (Figure 6). There were no significant effects of OEA during acquisition, extinction, or re‐acquisition (all *ps* > .05), but there was a significant main effect during renewal, *F*(1,91) = 5.04, *p* = .027, ${\eta_{p}}^{2}$ = .05. Higher OEA was associated with lower TIRs during renewal.

## 3 DISCUSSION

The current study tested whether the use of a scream US produced fear conditioning similar compared to a shock US. Although participants in the Scream group reported higher experimentally induced anxiety, SCRs were significantly lower during acquisition and there was significantly less CS+/CS− differentiation for the Scream group compared to the Shock group. The Scream group also showed rapid habituation to the US during acquisition when compared to the Shock group. We also tested whether varying between a scream and shock US increased fear conditioning compared to using a scream or shock alone. In most cases, the Shock/Scream group displayed similar, though slightly lower, overall SCRs, and differential SCRs comparable to the Shock group, though the Shock/Scream group showed resistance to habituation toward the US compared to the Shock group. Finally, we tested the relationship between hair endocannabinoid levels and fear conditioning using exploratory analyses (due to low sample sizes). We found some evidence of lower renewal of conditioned fear associated with higher 2‐AG hair concentrations, however, these results were marginal and limited to third‐interval responses. Higher 2‐AG and OEA, as well as lower AEA, were associated with reduced overall SCRs during extinction and renewal. These findings have implications for design of fear conditioning experiments and provide evidence for a relationship between hair endocannabinoids, fear conditioning, and skin conductance responding.

Following the recent global challenges to in‐person research, fear conditioning research has begun to move online (McGregor et al., 2021; Purves et al., 2019). However, there has been little research on the difference in the effect during fear conditioning of auditory compared to electrotactile USs, which are usually used in the laboratory. In the current study, we found that, although the subjective ratings (i.e., CS pleasantness and arousal) were mostly comparable between the US types, SCRs in response to the CS + s in the Shock as well as Shock/Scream groups were significantly larger compared to the Scream group. Further to this, the Scream group showed substantial habituation to the US compared to the Shock and Shock/Scream groups during acquisition. Habituation to shock USs is reported in the literature as being associated with brainstem and hypothalamus activity (Peters et al., 2014; Spoormaker et al., 2010). Our findings are consistent with those of Glenn et al. (2012), who compared a human scream to electric shock but are not similar to Neumann and Waters (2006), who reported that a loud fork scraping noise produced SCR and heart rate fear conditioning comparable to an electric shock as well as a loud tone burst. Our results are also at odds with Sperl et al. (2016) who reported that a loud tone burst resulted in stronger conditioning (i.e., higher acquisition and impaired extinction) compared to a shock over many trials. However, it should be noted that the latter study used a longer conditioning paradigm compared to our own with 45 trials and 4 CSs during acquisition and 50 trials during extinction, and Neumann and Waters (2006) did not use a scream but rather a scraping noise, which could explain the differences between studies. It should also be noted that our Scream group showed lower electrodermal FIRs on the first block of habituation; this is likely because they did not undergo a shock workup, which has been shown to increase response during habituation (Lipp et al., 2015).

Interestingly, no study to our knowledge has used more than one type of US during conditioning. We had hypothesized that, due to the decrease in predictability of the type of US that would be experienced, participants in the Shock/Scream group would show higher anticipatory responding compared to the Shock and Scream alone groups. This hypothesis was made on the basis that fear—as well as extinction—learning rests on the premise that expectancy violation (akin to unpredictability of outcome) is a strong driver of learning (Craske et al., 2014; Rescorla & Wagner, 1972). Our results showed some evidence for the effectiveness of the manipulation—both during acquisition and re‐acquisition, unconditioned responding (i.e., SCRs during the TIR) did not habituate in the Shock/Scream group when compared to the Scream or Shock groups. However, both differential and non‐differential FIRs, SIRs, and subjective ratings in the Shock/Scream condition were either similar to or lower than those observed in the Shock condition. This finding implies that making the nature of the US unpredictable did not make the associated CS a more ‘feared’ stimulus. Importantly, most of the between group differences in this study were found in the second and third interval responses, which are more associated with US anticipation and response to the US, respectively (Prokasy & Ebel, 1967). In comparison, the FIR which is typically the primary dependent measure considered in fear conditioning literature (Lonsdorf et al., 2017) is more associated with orienting responses to CS onset. The use of SIRs and TIRs in this study allowed us to identify differential reactivity between the groups that a focus on FIRs would have missed. These findings suggest that anticipatory or preparatory responses differ as a function of the nature of the US.

In the current study, hair endocannabinoid concentrations were measured using mass spectrometry and correlated with fear conditioning throughout the experiment. Based on previous studies, we expected that higher levels of AEA and 2‐AG would be associated with better fear extinction and lower return of fear (Hill et al., 2018; Marsicano et al., 2002; Morena et al., 2016; Ney, Matthews, et al., 2021). We found partial support for this hypothesis in that higher 2‐AG was associated with lower return of fear, but only during TIRs in renewal, suggesting that this effect was associated only with reactivity toward the absence of the shock rather than shock anticipation (SIR) or CS orienting (FIR). There were no significant effects of AEA. These findings can be explained in a number of ways. Firstly, previous studies have strongly suggested that activation of the cannabinoid receptor 1 is necessary for fear extinction to occur (Akirav, 2011; Chhatwal et al., 2005; Marsicano et al., 2002). As 2‐AG is a cannabinoid receptor agonist, we would expect that higher concentrations would be associated with better extinction learning and subsequently lower return of fear. Although a similar finding was expected for AEA, our previous work identified subpopulations of participants (those with higher posttraumatic stress symptomology) that seemed to display higher fear conditioning if they had higher AEA (Ney, Matthews, et al., 2021). It is possible that AEA is a less reliable marker for fear conditioning than was previously anticipated. However, it is also very probable that our analyses for AEA and 2‐AG were underpowered, given the technical difficulties we experienced during sample processing, and that this issue may have been resolved through acute measurement (e.g., blood samples) and perhaps hair sampling is not the ideal approach. Ultimately, these findings therefore require replication before any firm conclusions can be drawn. Higher hair AEA was associated with higher FIRs during extinction, except in the first Block where higher hair AEA was associated with lower FIRs. Similarly, higher hair AEA was associated with higher SIRs throughout extinction. Higher 2‐AG was associated with reduced overall electrodermal FIRs during extinction, but not acquisition, renewal, or reacquisition. There were significant effects of OEA on SIRs and TIRs during renewal. Since none of these effects were moderated by CS type, it can only be concluded that these effects reflected an overall association between these endocannabinoid levels and physiological arousal, rather than conditional learning. Associations between OEA and differential fear conditioning were not found in the current experiment, which was expected given that OEA does not interface with the cannabinoid type 1 receptor (Ligresti et al., 2016). As stated above, these results should be considered exploratory given the low number of participants included. Finally, skin conductance primarily indexes arousal and contingency learning during fear conditioning (Lipp, 2006). Future studies should also investigate whether the endocannabinoid findings in this study generalize to more valence‐specific measures of fear conditioning, such as startle reactivity. Our study had several limitations that should be considered. Firstly, the Scream group did not have a shock workup procedure, and this clearly impacted SCRs during habituation. It is unknown whether the absence of a shock workup affected the results in the rest of the study, and although previous research suggests that this effect is likely limited to habituation (Lipp et al., 2015), we cannot rule out the possibility that acquisition differed between groups due to the presence or absence of a shock workup prior to habituation. Secondly, due to loss of instrument sensitivity and significant delays in sample processing, only one‐third of the hair samples had quantifiable levels of AEA and 2‐AG. The main reason for this issue was loss of mass spectrometry sensitivity, which was evidenced by the surrogate labeled standards showing substantially lower sensitivity across all samples compared to runs in the recent past. 2‐AG and AEA were quantifiable less often than OEA because OEA is far more abundant that 2‐AG or AEA in this sample matrix, so the loss in sensitivity was only an issue for low abundant targets, with 2‐AG and AEA being relatively low abundance in hair. This means that our biological analyses lacked statistical power and will need replication with larger samples. Future studies should analyze samples within 6–12 months of collection. Participants scored higher than a normative sample on the PSWQ, which is a measure of trait worry (Meyer et al., 1990). We are unsure whether our findings would generalize to a sample that has lower levels of worry. Unfortunately, we were unable to assess the perceived US intensity and pleasantness across groups due to a programming error. Finally, the groups had slightly different sample sizes, sometimes resulting in 13% more participants in the Shock group compared to the Scream group (e.g., habituation).

In conclusion, we found significant differences in conditioned electrodermal SIRs and TIRs between shock, scream, and variable shock/scream USs. These differences indicate that responses to a scream US habituate rapidly and that it supports lower differential conditioning, as well as overall physiological arousal, in the SIR and TIRs of acquisition, renewal, and re‐acquisition phases, but not extinction. A variable shock/scream US schedule performs similarly to one using only a shock US but with slightly lower differential and overall responding, as well as reduced habituation during acquisition and re‐acquisition. Finally, we found some evidence that hair endocannabinoids affect the renewal of conditioned fear, as well as overall physiological arousal during fear conditioning. These findings strongly suggest that different USs produce substantially different anticipatory conditioned responses and that these responses have different characteristics throughout fear conditioning paradigms. When using SCRs, where possible shocks should be the preferred US due to higher conditioned responding. More research using larger samples is needed to examine the relationship between hair endocannabinoids and fear conditioning, though our exploratory work here suggests that 2‐AG levels may be associated with conditioned fear renewal.

## AUTHOR CONTRIBUTIONS

**Luke J. Ney:** Conceptualization; data curation; formal analysis; investigation; methodology; project administration; software; supervision; visualization; writing – original draft; writing – review and editing. **David Nichols:** Conceptualization; formal analysis; investigation; resources; software; supervision; writing – review and editing. **Ottmar V. Lipp:** Conceptualization; data curation; funding acquisition; methodology; resources; software; supervision; writing – review and editing.

## FUNDING INFORMATION

This work was supported by the Australian Research Council (DP180100869) and the National Health and Medical Research Council (APP1156490).

## Data Availability

The data that support the findings of this study are available from the corresponding author upon reasonable request.
